# Adjustable delivery of pro-angiogenic FGF-2 by alginate:collagen microspheres

**DOI:** 10.1242/bio.027060

**Published:** 2018-02-15

**Authors:** Zaheer Ali, Anik Islam, Peter Sherrell, Mark Le-Moine, Georgios Lolas, Konstantinos Syrigos, Mehrdad Rafat, Lasse D. Jensen

**Affiliations:** 1 Department of Medical and Health Sciences, Division of Cardiovascular Medicine, Linköping University, Linköping SE-58183, Sweden; 2 Department of Materials, Faculty of Engineering, Imperial College London, London, SW7 2AZ, United Kingdom; 3 Department of Biomedical Engineering, Linkoping University, Linköping SE-58183, Sweden; 4 Oncology Unit, 3rd Department of Medicine, ‘Sotiria’ General Hospital, National and Kapodistrian University of Athens, Athens 115 27, Greece

**Keywords:** Hydrogels, Microspheres, Angiogenesis, Vasculature, Zebrafish

## Abstract

Therapeutic induction of blood vessel growth (angiogenesis) in ischemic tissues holds great potential for treatment of myocardial infarction and stroke. Achieving sustained angiogenesis and vascular maturation has, however, been highly challenging. Here, we demonstrate that alginate:collagen hydrogels containing therapeutic, pro-angiogenic FGF-2, and formulated as microspheres, is a promising and clinically relevant vehicle for therapeutic angiogenesis. By titrating the amount of readily dissolvable and degradable collagen with more slowly degradable alginate in the hydrogel mixture, the degradation rates of the biomaterial controlling the release kinetics of embedded pro-angiogenic FGF-2 can be adjusted. Furthermore, we elaborate a microsphere synthesis protocol allowing accurate control over sphere size, also a critical determinant of degradation/release rate. As expected, alginate:collagen microspheres were completely biocompatible and did not cause any adverse reactions when injected in mice. Importantly, the amount of pro-angiogenic FGF-2 released from such microspheres led to robust induction of angiogenesis in zebrafish embryos similar to that achieved by injecting FGF-2-releasing cells. These findings highlight the use of microspheres constructed from alginate:collagen hydrogels as a promising and clinically relevant delivery system for pro-angiogenic therapy.

## Abstract (precis)

**Summary:** The development of alginate:collagen composite hydrogel microspheres of adjustable size and degradation speed is described as a new platform for delivery of pro-angiogenic FGF-2 or pro-angiogenic cells.

## INTRODUCTION

Insufficient perfusion of blood through tissues drives tissue damage and death in myocardial infarction (MI), stroke or other ischemic disorders constituting the leading cause of mortality and morbidity (Eltzschig et al., 2011). In order to treat these diseases, much effort has been placed on avoiding ischemia-induced cell death and assisting the regenerative process by various types of tissue engineering approaches using biocompatible materials (Emmert et al., 2014). Both of these processes require therapeutic induction of new vessel growth (angiogenesis) into the affected tissue (Cao, 2010). Despite the unparalleled clinical importance of effective therapeutic angiogenesis regimens especially for MI and stroke patients, there are currently no approved methods available to accomplish this.

The lack of clinical success in this area may in part be due to problems in delivery and retention of therapeutic, pro-angiogenic cells or growth factors in the ischemic tissue (Cao, 2010; Rufaihah and Seliktar, 2010). Delivery regimens should preferably achieve sustained, high and local concentrations of therapeutic cells or growth factors, preferably in a minimally invasive manner such as by delivery via a catheter brought into the coronary or cerebral circulation via the femoral vein. Formulating the injectable treatment for this purpose is, however, highly challenging as aqueous suspensions of cells or growth factors have suffered from poor retention of the therapeutics in the ischemic tissue and therefore provide poor support for tissue regeneration (Gelmi et al., 2016).

A way to combat such issues has been to incorporate the therapeutic agent into biocompatible hydrogel scaffolds that combine sustained release of cells or growth factors with good retention of the hydrogel in the tissue, while at the same time providing a matrix which supports the survival, migration and differentiation of the regenerating cells, thus improving the regeneration of the damaged tissue (Emmert et al., 2014; Gelmi et al., 2016). For MI-applications, such materials must be able to withstand the mechanical forces exerted on the myocardium due to constant cardiac contractions, while also maintaining adherence to the damaged tissue and be sufficiently elastic to follow the cardiac movements without resistance. Hydrogels are fully biocompatible materials which may be engineered to exhibit the desired degradation properties, hardness, or binding strength to therapeutic drugs imbedded in the material and tissues to which the hydrogels are delivered, leading to higher tissue tolerance and retention compared to other materials classically used for delivery of proangiogenic therapies (Mayfield et al., 2014). The delivery of the hydrogel is, however, challenging when prepared as highly viscous formulations that are not suitable for injection through low-caliber catheters, and require direct intra-myocardial injection (Gelmi et al., 2016; Mayfield et al., 2014), a procedure that necessitates major surgery and is associated with significant risk for the patient.

Collagen is a highly attractive bio-polymer that has been studied for use in corneal (Rafat et al., 2016), bone (Sarker et al., 2015; Pina et al., 2015), cartilage (Puetzer and Bonassar, 2016) and cardiac (Tallawi et al., 2015) tissue engineering due to the high biocompatibility, enzymatic degradability, tunable mechanical strength, and flexibility of fabrication methods (Khorshidi et al., 2015). Furthermore, collagen has good miscibility with a variety of other bio-polymers allowing further tailoring of fabrication methods, stiffness, degradability, water-content, and chemical functionality of the tissue scaffold (Khorshidi et al., 2015; Ayala et al., 2015). The combination of collagen with a more slowly degradable ionically cross-linked bio-polymer, alginate, also opens up key pathways for adjustable drug release kinetics, and rapid scaffold fabrication and disintegration (Ayala et al., 2015).

Recently, we have provided proof of principle for such an alternative approach, the formulation of the hydrogel vehicle as microspheres, small enough to pass through catheters for percutaneous, transarterial delivery and due to their geometry and relative stiffness, cope very well with the high mechanical strain in the contracting myocardium (Sherrell et al., 2016). The stability of these microspheres depended on their relative concentration of alginate and collagen; collagen-rich microspheres were less stable in aqueous solutions compared to alginate-rich microspheres. Here we show that alginate:collagen mixtures allow for sustained release of pro-angiogenic FGF-2 added to the materials directly or produced by embedded cells, which elicit robust angiogenic responses *in vivo* using zebrafish embryos. Furthermore, we show that these alginate:collagen spheres are biocompatible and do not lead to inflammation or other host reactions when injected into mice. We provide a protocol for adjusting the size and cell density of the spheres during their production to allow adjustment of the amount of growth factor or cells incorporated into the spheres and the kinetics of their release. These findings, therefore, establish alginate:collagen hydrogels, formulated as microspheres, as a highly versatile and promising vehicle for delivery of therapeutic pro-angiogenic FGF-2 for treatment of ischemic disorders including MI and stroke.

## RESULTS

### Adjustable hydrogel degradation kinetics by mixing alginate and collagen

**Fig. 1.**
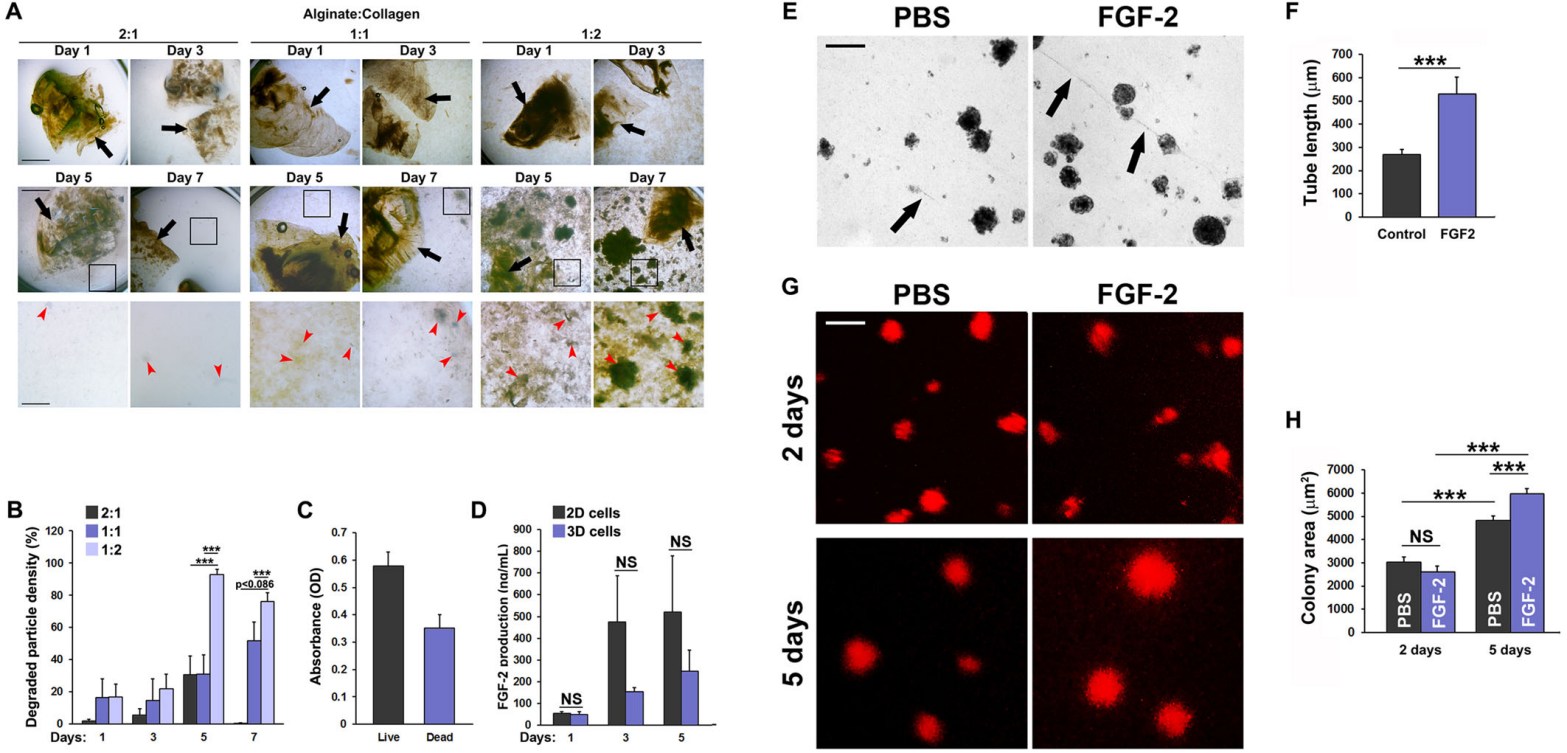
**Alginate:collagen hydrogels support controlled release of therapeutic factors and cell viability *in vitro*.** (A) Bright field micrographs of 2:1, 1:1 or 1:2 mixtures of alginate:collagen hydrogel patches incubated in cell growth medium at 37°C for 1-7 days. Black arrows indicate intact hydrogel pieces. Red arrowheads indicate pieces of degraded hydrogel material, black boxes indicate the region shown in the enlarge image below the overview image. Scale bars: 1000 µm in the first two rows and 500 µm in the third row. (B) Quantification of the mean cumulative degradation (density of degraded hydrogel particles) of the hydrogel patches after 1, 3, 5 and 7 days of incubation. 2:1, 1:1 and 1:2 indicate the relative concentrations of alginate to collagen. Error bars indicate s.e.m. ***P<0.001. *n*=7. (C) Quantification of the mean absorbance of dyes labeling living versus dead K1000 cells following overnight incubation embedded in 1:1 alginate:collagen hydrogel patches. *n*=12. (D) ELISA quantification of free hFGF2 in the medium of 1:1 alginate:collagen hydrogel patches containing 10^6^ K1000 cells and incubated for 1-5 days in DMEM growth medium at 37°C. Error bars indicate s.e.m. *n*=4. (E) Bright field micrographs of 1:1 alginate:collagen hydrogel patches made with PBS or 1000 ng/ml FGF2, 6 days after 10^6^ PAECs were seeded onto their surface and incubated in DMEM growth medium at 37°C. Black arrows point to PAEC tube-like structures. Scale bar: 100 µm. (F) Quantification of the length of tubes as indicated in C. ***P<0.001, *n*=8. (G) Fluorescent micrographs of DiI-labeled PAECs grown for 2-5 days in DMEM at 37°C on 1:1 alginate:collagen hydrogel patches made with PBS or 1000 ng/ml FGF2. Scale bar: 100 µm. (H) Quantification of the area of the red colonies shown in E. ***P<0.001, *n*=115, 124, 309, 295 colonies were counted from six images in the 2 days PBS, 2 days FGF-2, 5 days PBS and 5 days FGF-2 groups, respectively.

Implantable biomaterials for treatment of ischemic disorders should preferably provide a platform for sustained stimulation of the regenerative process, but eventually degrade to avoid retention of large amounts of artificial, non-self-components that may impair restoration of normal tissue functions. To characterize the degradation kinetics of alginate:collagen-based biomaterials, we generated hydrogels consisting of 2:1-, 1:1- or 1:2-fold mixtures of alginate and collagen. In line with previously reported results on the alginate:collagen system (Sherrell et al., 2016), hydrogels generated using twice as much alginate as collagen (i.e. 2:1 mixture) were relatively stable in Dulbecco's modified Eagle medium (DMEM) medium at 37°C, with only very little degraded hydrogel particles visible in the medium after 7 days of incubation (Fig. 1A). In contrast, the hydrogels containing double the amount of collagen relative to alginate (1:2 mixture) had started disintegrating already after 1 day of incubation (Fig. 1A,B) and were severely disintegrated after 5 days, with little intact gel-material remaining in the solution (Fig. 1A,B). As expected, hydrogels containing equal amount of alginate and collagen (1:1 mixture) exhibited intermediate stability, having disintegrated significantly after 7 days of incubation, but also with significant amounts of gel-material still intact (Fig. 1A,B). Due to the moderate degradation kinetics, we chose the 1:1 alginate:collagen hydrogel mixture for further experimentation.

### Alginate:collagen hydrogels enable pro-angiogenic functions of embedded therapeutic cells

For therapeutic purposes, hydrogels must be able to sustain cell functions and enable therapeutic cells to produce pro-angiogenic and regenerative factors (Rufaihah and Seliktar, 2010). To test if the alginate:collagen scaffold were suitable for such cell-based therapies, we embedded FGF-2-producing K1000 fibroblasts into hydrogel patches and analyzed their viability and the kinetics of FGF-2 release into the medium over time. We observed that almost 2/3 of the cells were still viable after embedding within the hydrogels (Fig. 1C). Importantly, viable, embedded K1000 cells started producing FGF-2 which could be detected in medium already after the first day of culture. Alginate contains proteoglycan motifs which resemble those in extracellular matrix (ECM) proteins and therefore bind angiogenic factors to a similar extent as the ECM. In order to analyze the retention/release of K1000-derived FGF-2, one of the most strongly ECM-binding angiogenic factors, we analyzed the release kinetics of embedded cells versus non-embedded cells growing in 2D, over time, by enzyme-linked immunosorbent assay (ELISA). The levels of FGF-2 found in the medium increased significantly from one to three days of culture, and even further by the fifth day of culture, especially in the embedded cells growing in 3D group (Fig. 1D). This is likely a result of the increased degradation of the biomaterial at this time-point (Fig. 1B). Taken together, these results indicate that the cells were metabolically and transcriptionally active within the material and that the hydrogel augmented the release of FGF-2 over time, with a kinetic profile similar to its degradation rate (Fig. 1B,D). Cells embedded in 3D within the hydrogel exhibited a trend towards releasing less FGF-2 compared to cells growing without hydrogel in 2D, but this was not statistically significant (Fig. 1D). The slightly lower release of FGF-2 by the embedded cells could however be explained by the likely sequestration of part of the produced FGF-2 within the non-degraded part of the hydrogel.

The hydrogel must also provide a suitable framework for growth and maintenance of new vessels (Rufaihah and Seliktar, 2016). Endothelial cells such as porcine aortic endothelial cells (PAECs) spontaneously reorganize into tube-like vascular structures when grown on a suitable matrix *in vitro* (Bayat et al., 2015). In order to investigate if alginate:collagen hydrogels allow for such pro-angiogenic behavior, we added PAECs to 1:1 alginate:collagen hydrogels with or without FGF-2. PAECs, first organized into clusters of endothelial cells, which, in some cases on FGF-2-containing hydrogels, extended tube-like vascular structures from one cluster to the next. PAECs seeded on hydrogel without FGF-2 also formed colonies and a few short, tube-like structures, which, however, rarely extended beyond two adjacent PAEC clusters (Fig. 1E,F). PAECs labeled with the red-fluorescent membrane dye 1,1′-Dioctadecyl-3,3,3′,3′-tetramethylindocarbocyanine perchlorate (DiI, Sigma-Aldrich), to facilitate visualization of the cells, readily proliferated leading to a growth in the cluster size between 2 and 5 days post cell seeding both under normal conditions but more so when the hydrogel patch was laced with FGF-2 (Fig. 1G,H). Combined, these findings indicate that alginate:collagen hydrogels are good vehicles for controlled release and delivery of pro-angiogenic FGF-2 or FGF-2-producing cells and that they support proliferation and spontaneous vessel formation of endothelial cells.

### Encapsulation of cells in alginate:collagen microspheres is mainly regulated by sphere diameter

**Fig. 2.**
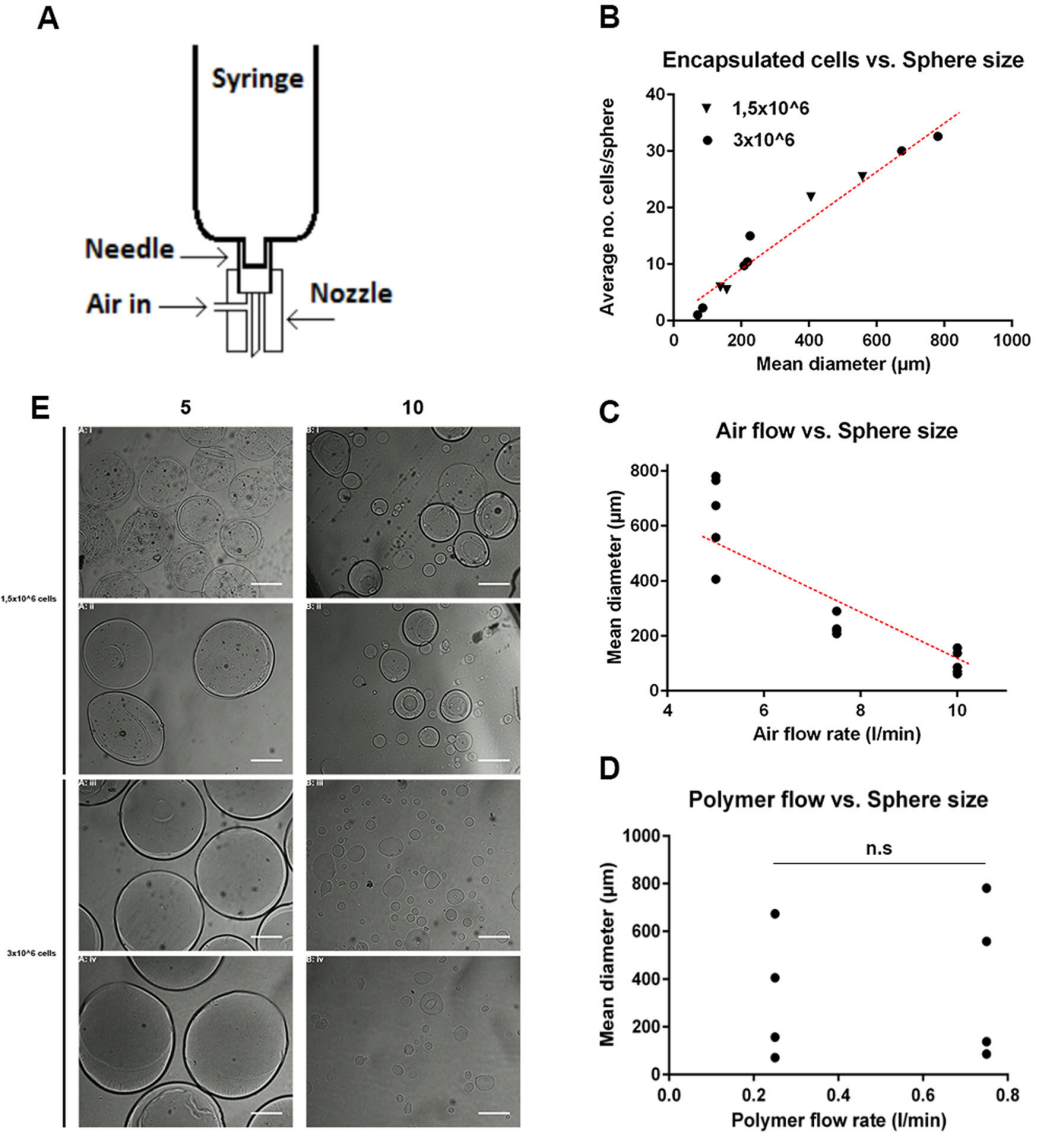
**Synthesis of alginate:collagen microspheres.** (A) Schematic representation of the set-up for synthesis of alginate:collagen hydrogel microspheres. (B) Quantification of and correlation between the number of cells encapsulated and diameter of the microspheres synthesized using the 1:1 alginate:collagen polymer mix under various parameters as indicated in Table 1. Black triangles represent the addition of 1.5×10^6^ cells to the 10 ml polymer solution, black circles represent the addition of 3.0×10^6^ cells to the 10 ml synthesis polymer solution. Each value represents the mean of at least 20 individual microspheres from a separate synthesis experiment. (C) Quantification of the mean diameter of spheres synthesized using the 1:1 alginate:collagen polymer mix and different air flow-rates as indicated in Table 1. Each value represents the mean of at least 20 individual microspheres from a separate synthesis experiment. (D) Quantification of the mean diameter of spheres synthesized using the 1:1 alginate:collagen polymer mix and different polymer flow-rates as indicated in Table 1. Each value represents the mean of at least 20 individual microspheres from a separate synthesis experiment. n.s., not significant. (E) Bright field micrographs of spheres containing cells synthesized using air flow-rates of 5 l/min (left) or 10 l/min (right), polymer flow rates of 0.25 l/min (first and third row) or 0.75 l/min (second and fourth row) or using 1.5×10^6^ cells (upper four images) or 3.0×10^6^ cells (lower four images). Scale bars: 50 µm. Dark dots inside the microspheres are indicative of cells.

Compared to crude solutions of hydrogels, formulating hydrogels as microspheres increases the tensile strength of the material, increases injectability/reduces overall viscosity, and reduces the forces exerted by the material on the tissue (Leslie et al., 2013). Increasing the surface-to-volume ratio in this way may also improve the release-profile of embedded cells or drugs and the clearing of slowly degradable alginate remnants once the biomaterial has done its job. Production of alginate:collagen microspheres can be achieved by pressing the hydrogel through a nozzle coupled to an air-jet followed by calcium-mediated crosslinking of the alginate scaffolds of the resulting droplets (Fig. 2A). We have recently shown that this technique allows the generation of microsphere-cocooned cell formulations for therapeutic applications (Sherrell et al., 2016), but the extent to which the cell-content in the microspheres can be controlled is not known. As the diameter and number of cells per volume hydrogel are key parameters for determining the amount of cells and/or growth factors embedded in the hydrogel that are delivered to the tissue, we sought to develop a protocol in which these parameters could be accurately controlled by adjusting cell concentration in the hydrogel polymer, the shear forces applied to the polymer jet during sphere synthesis, and the volume of material ejected per second (air flow rate and polymer flow rate respectively). In our set-up, the number of cells per synthesized microsphere was tunable between 1±1 cell/sphere and 33±9 cells/sphere (Table 1 and Fig. 2B). Adjusting the cell content to the average volume of the microspheres generated under each production protocol, and taking the encapsulation percentage into account (Table 1), this corresponds to between 0.12±0.05 and 32±32 cells per nl of microsphere/biomaterial. Surprisingly, the number of cells per sphere was shown to be linear with mean sphere diameter regardless of the concentration of cells in the liquid, non-cross-linked polymer solution (Fig. 2B). These results indicate, based on the cubic relation between radius and volume, that smaller spheres give rise to the delivery of more cells per volume of the hydrogel compared to larger spheres (Table 1).

**Table 1.**
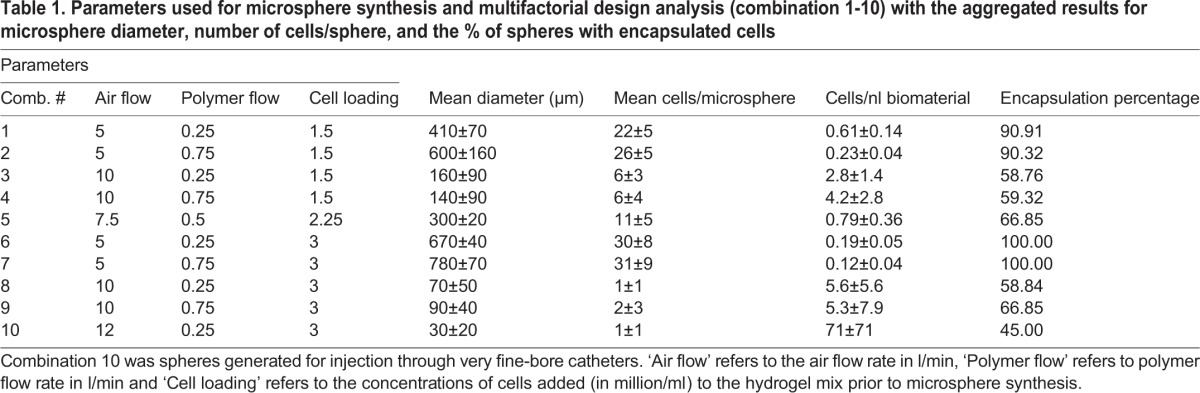
**Parameters used for microsphere synthesis and multifactorial design analysis (combination 1-10) with the aggregated results for microsphere diameter, number of cells/sphere, and the % of spheres with encapsulated cells**

### Air flow rate during sphere synthesis is the most important parameter for determining sphere diameter and number of encapsulated cells per volume hydrogel

In order to analyze the interactions between the synthesis parameters: cell concentration, air flow rate and polymer flow rate, and the examined responses: sphere diameter, number of cells per sphere, and the percentage of spheres containing encapsulated cells, we developed a multivariate experimental design approach (Table 2), where such interactions could be analyzed quantitatively and their significance could be statistically tested. We found that all three synthesis parameters (X_1_, X_2_ and X_3_ denoting cell concentration, air flow rate and polymer flow rate respectively) have a direct and significant effect on the produced sphere diameter, with air flow rate and polymer flow rate following the expected trends for sphere size, as given by the sign on the co-efficient (higher air flow rate and lower polymer flow rate leading to smaller microspheres). The magnitude of the coefficients, however, demonstrate that the range of air flow rates examined (5 l/min to 10 l/min) have an effect magnitude of approximately six times that of both cell concentration (1.5 million cells/ml to 3 million cells/ml) and polymer flow rate (0.25 ml/min to 0.75 ml/min), and, as such, is much more important for determining sphere diameter and thus number of cells per nl of biomaterial (Fig. 2C,E). In fact, polymer flow rate only exhibited significant effects on sphere diameter in the factorial design, not while evaluating the mean diameter of spheres at two different polymer flow rates independently (Fig. 2D,E). These conflicting statistical results may be explained by the much more numerous degrees of freedom inherent in the factorial design compared to testing between two isolated groups.

**Table 2.**
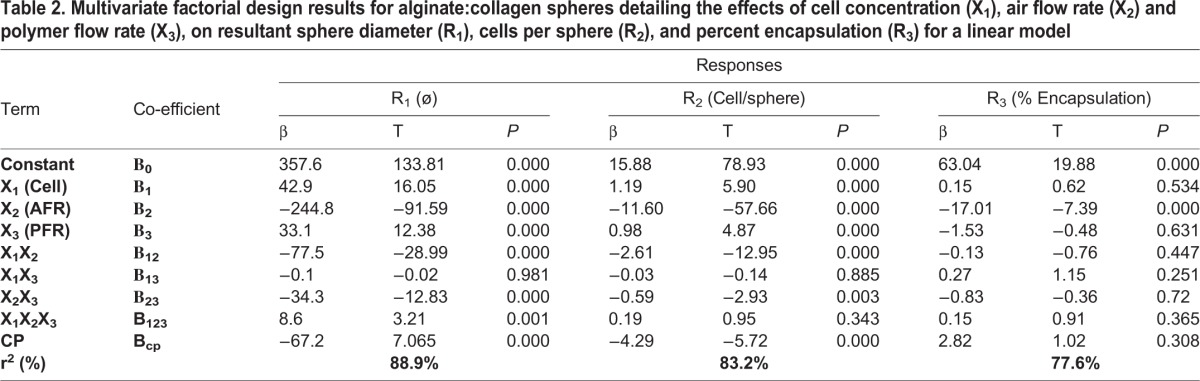
**Multivariate factorial design results for alginate:collagen spheres detailing the effects of cell concentration (X_1_), air flow rate (X_2_) and polymer flow rate (X_3_), on resultant sphere diameter (R_1_), cells per sphere (R_2_), and percent encapsulation (R_3_) for a linear model**

### Customized design of hydrogel microspheres with desired characteristics

By using the factorial model we set up a quantitative regimen for the production of spheres at a desired diameter, with a desired number of cells per sphere and a desired number of spheres containing cells using the simultaneous equations (X_1_, X_2_ and X_3_ range between −1 and 1 as arbitrary units given these constants):

R_nø_=357.6+42.9X_1_–244.8X_2_+33.1X_3_–77.5X_1_X_2_–0.1X_1_X_3_–34.3X_2_X_3_+8.6X_1_X_2_X_3_

R_ncells_=15.88+1.19X_1_–11.6X_2_+0.98X_3_–2.61X_1_X_2_–0.03X_1_X_3_–0.59X_2_X_3_+0.19X_1_X_2_X_3_

R_nencapsulation_=63.04+0.15X_1_–17.01X_2_–1.53X_3_–1.63X_1_X_2_+0.27X_1_X_3_–0.83X_2_X_3_+0.15X_1_X_2_X_3_

These equations demonstrate the parameters required to synthesize microspheres for injection through very fine-bore catheters, that is, spheres with a diameter of approximately 30 μm, with a maximum loading of cells per sphere and maximum number of spheres containing cells, would be to use an exceptionally low polymer flow rate, high air flow rate and high cell concentration. Using a polymer flow-rate of 0.25 ml/min and a cell concentration of 3 million cells/ml, the air flow rate required for the synthesized microspheres to be 30 μm was found to be 12 l/min (Table 1). As calculated, using a polymer flow-rate of 0.25 ml/min, 3 million cells/ml and an air flow rate of 12 l/min indeed resulted in the synthesis of microspheres with a diameter of 30±20 mm containing 0-2 cells per microsphere and with an encapsulation efficiency of approximately 50% (Table 1). These optimal conditions furthermore produced microspheres with the highest density of cells per volume hydrogel (approximately 32 cells/nl).

### Release of FGF-2 or cells from alginate:collagen microspheres

**Fig. 3.**
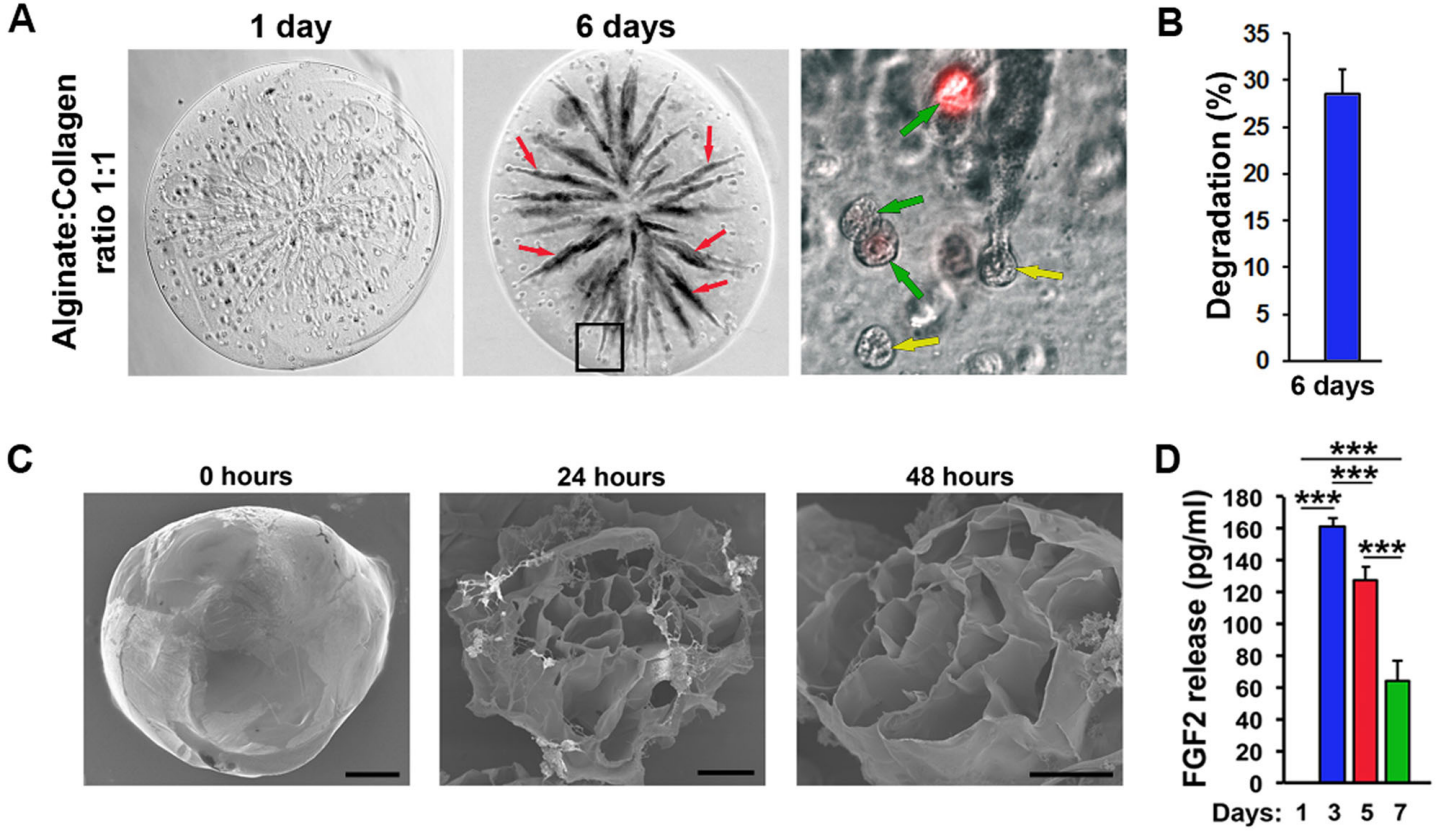
**Release of therapeutic cells or factors from alginate:collagen microspheres.** (A) Bright field and fluorescent micrographs of 1:1 alginate:collagen spheres containing DiI-labeled PAECs (red in the image to the right) 1-6 days after synthesis incubated in DMEM growth medium. Red arrows indicate tunnels created from dissolution of collagen and movement of cells through the microsphere. Green arrows indicate DiI-positive PAECs and yellow arrows indicate DiI-negative PAECs. Black box in the middle image indicates the region enlarged in the image to the right. (B) Quantification of the proportion of dark tunnels as indicated with red arrows in A versus the whole microsphere area 6 days after synthesis. *n*=8. (C) TEM micrographs of closed (left image) or opened (middle and right-most images) microspheres immediately (left image), 24 h (middle image) or 48 h (right image) after synthesis and incubation at 37°C. Scale bars: 100 µm. (D) ELISA quantification of the amount of FGF2 released from microspheres produced with 1:1 alginate:collagen containing 1000 ng/ml FGF2 after incubation in DMEM growth medium for 1-7 days. ***P<0.001, *n*=4.

Next, we analyzed the degradation kinetics of alginate:collagen microspheres. After 6 days at 37°C, the microspheres were partially degraded as evidenced by the clearly visible emergence of collapsed collagen-free tunnels, which appeared dark, using phase-contrast microscopy (Fig. 3A,B). Encapsulated cells stained with membrane-targeted dye (DiI) appeared healthy inside the spheres even 6 days after synthesis (Fig. 3A). Considering that hydrogel-encapsulated cells are still metabolically active and produce pro-angiogenic factors (Fig. 1C,D), these results indicate that embedding such cells into microspheres may be a viable strategy for improved delivery to ischemic tissues. Electron microscopic (EM) evaluation of the early degradation of 1:1 alginate:collagen microspheres clearly shows an increased porosity (larger holes) in the cross-linked alginate matrix following 48 h, compared to 24 h at 37°C (Fig. 3C). Embedding FGF-2 into the microspheres similarly led to the release of this factor into the medium with a release-profile consisting of an early release of a large proportion of the contained factor followed by a decreasing but sustained release over the sphere-degradation period (Fig. 3D).

### Alginate:collagen microspheres are well tolerated *in vivo*

**Fig. 4.**
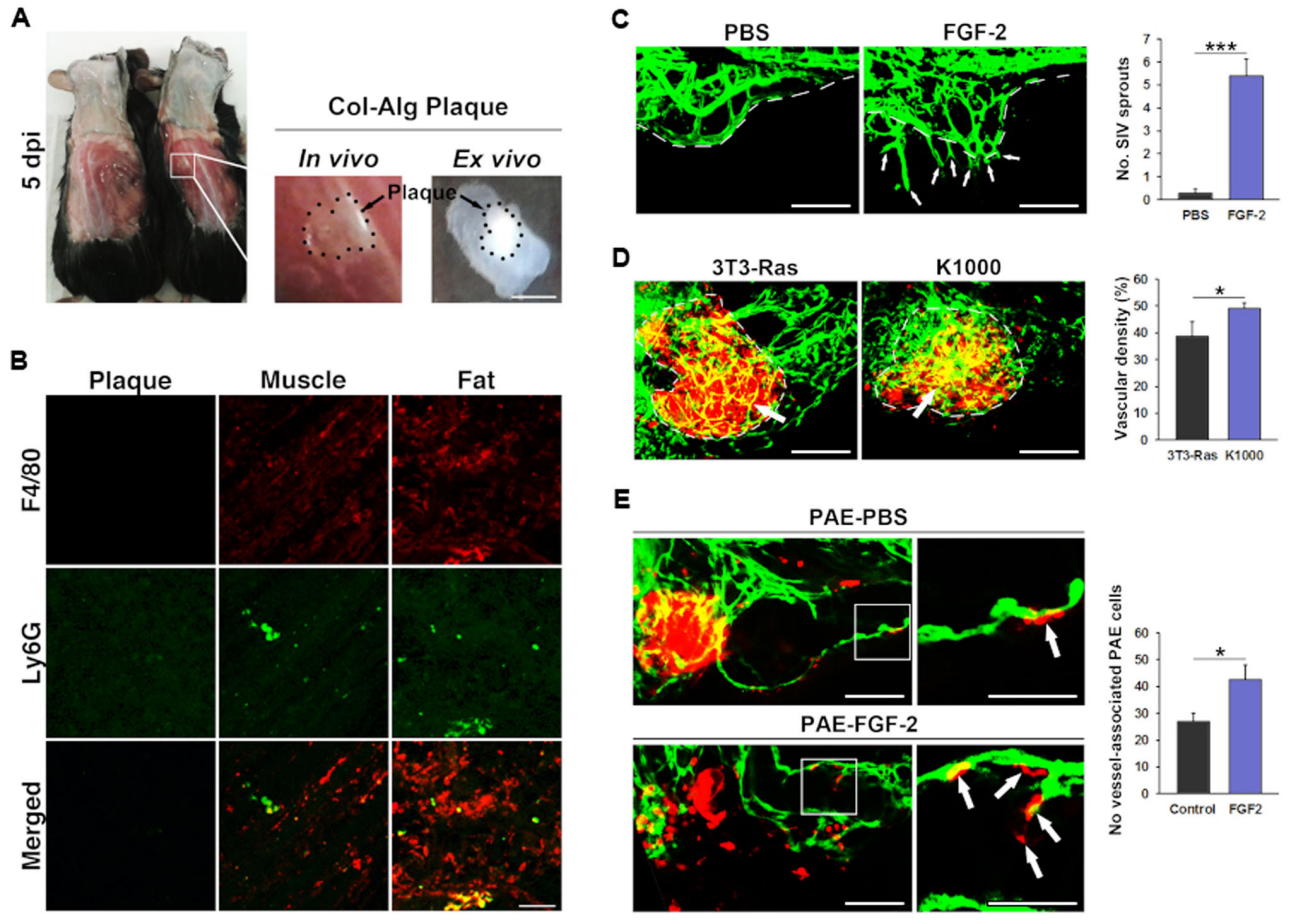
***In vivo* tolerance of alginate:collagen microspheres in mice and therapeutic angiogenesis in zebrafish.** (A) Photographs of mice (left and middle image) or excised hydrogel plugs (right image) 5 days after implantation of 10^5^ 1:1 alginate:collagen microspheres subcutaneously in 0.1 ml PBS. Scale bar: 500 µm. (B) Confocal micrographs of excised alginate:collagen plugs (left column) or adjacent muscle (middle column) or fat (left column), as shown in A, stained with antibodies against the pan-macrophage marker F4/80 (red, top row), the neutrophil marker Ly6G (green, middle row) or the merged images (bottom row). Scale bar: 50 µm. (C) Confocal micrographs of blood vessels (green) from fli1a:EGFP transgenic zebrafish embryos at 3 days post fertilization implanted with either PBS (left image) or 100 pg FGF-2 (left image) in the periviteline space 24 h prior. White dashed line indicates the sub-intestinal vessels. White arrows indicate FGF2-induced ectopic sprouts. The graph depicts the quantification of ectopic sprouts in PBS or FGF2-implanted zebrafish embryos shown in the images. ***P<0.001, *n*=12. Scale bar: 200 µm. (D) Confocal micrographs of blood vessels (green) from fli1a:EGFP transgenic zebrafish embryos at 5 days post fertilization implanted with either FGF2-non-producing 3T3-Ras cells (left image) or FGF2-producing K1000 cells (right image) in the periviteline space at 48 h post fertilization. White dashed line indicates the outline of the cell implants. White arrows indicate vessels that have grown into the cell implants. The graph depicts the quantification of vessel density in the cell implants from 3T3-Ras or K1000-baring zebrafish embryos shown in the images. *P<0.05, *n*=5. Scale bar: 200 µm. (E) Confocal micrographs of blood vessels (green) from fli1a:EGFP transgenic zebrafish embryos at 5 days post fertilization implanted with DiI-labeled PAECs (red) either in DMEM growth medium supplemented with vehicle (PBS, top row) or 100 pg/nl FGF2 (bottom row) in the periviteline space at 48 h post fertilization. White arrows indicate PAECs that have been incorporated into host blood vessels. White box in the images to the left indicate the region enlarged in the images to the right. The graph depicts the quantification of the number of DiI-labeled PAECs incorporated into host GFP-positive vessels in the PBS or FGF2 groups as shown in the images. *P<0.05, *n*=5. Scale bars: 200 µm, magnified images 50 µm.

In order to examine if alginate:collagen microspheres may lead to inflammation *in vivo*, we injected the microspheres subcutaneously into immune-competent C57/Bl6 mice and investigated the resulting microsphere plugs after 5 days. From macroscopic examination of the plugs, no obvious adverse reactions had occurred (Fig. 4A). Excising the plugs and performing immunohistochemistry using antibodies against the macrophage marker F4/80 and the neutrophil-marker Ly6G, we found no evidence of infiltration of macrophages or neutrophils into the plugs, although a physiological level of such cells were present in adjacent muscle or fat tissue (Fig. 4B). These findings do not exclude the possibility that adverse reactions to the hydrogels may arise later, nor that reactions taking place outside of the hydrogel local environment could lead to elevated levels of circulating inflammatory factors or cells, but indicate that the alginate:collagen microspheres do not cause acute, local immune cell infiltration inside the plaques, *in vivo*.

### Therapeutic levels of FGF-2 enhance angiogenesis *in vivo*

In order to test the biological effect of pro-angiogenic FGF-2 or therapeutic cells in concentrations found to be released from the hydrogels, we performed various *in vivo* experiments using zebrafish embryos to assay the effects on angiogenesis. In this assay, effects of pro-angiogenic factors or cells may be evaluated as their ability to induce sprouting and growth of the sub-intestinal vasculature (SIV), which develops between 2 and 3 days after fertilization of the egg. In agreement with the pro-angiogenic effects *in vitro* (Fig. 1E-H), we found that the levels of FGF-2 found in the medium 3 days after incubation of FGF-2-containing hydrogel microspheres elicited robust sprouting and growth of new GFP-tagged vessels from the ventral aspect of the SIV (Fig. 4C). Using the FGF-2 producing K1000 cells, which we found to be a sustained source of FGF-2 when encapsulated into the hydrogel microspheres, and non-FGF-2 producing 3T3-Ras fibroblasts as a control, we found that K1000 implants contained much higher vascular densities 3 days after implantation compared to 3T3-Ras implants (Fig. 4D). These findings indicate that the levels of FGF-2 mobilized from the hydrogels or FGF-2 producing cells give rise to robust angiogenic responses *in vivo*. In order to gain therapeutic benefit from adding endothelial cells to hydrogel scaffolds, the vascular structures formed within the scaffold also need to connect to the host vasculature. We tested this by injecting red fluorescently labeled PAECs with or without FGF-2 at the amount released by the hydrogel into zebrafish embryos with GFP-tagged vessels. Red fluorescent PAECs readily incorporated into green host blood vessels under both conditions, but the presence of FGF-2 led to a more efficient incorporation compared to when the cells were injected alone (Fig. 4E), indicating that exogenous endothelial cells may become part of an actively growing vasculature. These findings suggest that adding pro-angiogenic factors, such as FGF-2, and endothelial or endothelial progenitor cells to the hydrogel scaffolds would increase the re-vascularization of the host tissue.

## DISCUSSION

Here we report the design of hydrogels based on mixing readily degradable collagen with more slowly degradable, mesh-forming alginate polymers, and formulating the resulting hydrogels as injectable microspheres, allowing for adjustable release kinetics of pro-angiogenic FGF-2 or cells based on the concentration of collagen used and the diameter of the synthesized spheres. We provide a framework for producing spheres with diameters of 30 µm or higher, which would allow for local delivery of spheres to the ischemic site by percutaneous catheters. We show that encapsulated therapeutic cells survive, remain metabolically active, grow and undergo beneficial morphologic changes such as forming vascular tubes in this material at least for 1 week *in vitro*. Importantly, we show that the material is completely tolerated in mice and that local concentration of cells or factors achieved from delivery of such hydrogel microspheres induce therapeutic angiogenesis and coupling of therapeutic endothelial cells to the host vasculature in zebrafish.

The use of hydrogels as delivery vehicles for therapeutic cells or factors is, although very promising, still in its infancy. There is an urgent need to understand what hydrogel characteristics are most likely to give good engraftment and regenerative responses *in vivo* and use such information to design the best formulations of such products. Alginate and collagen are commonly used biological polymers in hydrogels (Rufaihah and Seliktar, 2010; Rafat et al., 2016; Leslie et al., 2013), due to their excellent biocompatibility and structural qualities which support tissue regeneration by host-cells. For example, composite collagen hydrogels demonstrating variable degradation characteristics have recently been engineered and used as artificial corneas, and have been shown to support the replacement of the artificial graft with host cells in a seamless transition over time (Rafat et al., 2016). Likewise, alginate hydrogels support efficient axon and peri-axonal cell growth and regeneration of the spinal cord in rats, especially when laden with therapeutic growth factors or cells (Günther et al., 2015). However, both collagen and alginate are also associated with drawbacks which limit their widespread application. Collagen is rather soluble in biological fluids and hydrogels/biomaterials consisting of only collagen will therefore disintegrate with a rate that depends on the hardness/compression of the collagen matrix (Rafat et al., 2016, 2008; Friess, 1998). Injectable collagen-only-based biomaterials are not sufficiently stable for supporting a matrix for regeneration of, for example, the myocardium. Indeed, we show here that hydrogels containing mostly collagen (i.e. double the amount of collagen compared to alginate) readily disintegrate in PBS over the course of less than 1 week. In contrast, alginate hydrogels are stable in biological fluids, and are degraded very slowly in the organism (Purcell et al., 2009). The optimal scaffold for regeneration would be one that may support tissue functions even during early regeneration, when few cells and matrices are present in the scaffold, but eventually become replaced by host cells and matrix proteins as these are produced during the regeneration process. Here we have shown that hydrogels containing a majority of alginate to collagen do not disintegrate over time, indicating that such materials may not be optimal as temporary scaffolds for regenerative therapy. We suggest that, depending on the application context, combinations of alginate and collagen may give improved control over the release of factors or cells from such matrices and combine stable and unstable biological scaffolds in a way that both secure fast recovery of tissue function as well as regeneration of near-physiological rather than artificial tissue. While we have found that degradation of such alginate:collagen mixtures can be adjusted depending on the amount of collagen used, how this relates to *in vivo* therapeutic angiogenesis, i.e. what release kinetics would be the most desirable for optimal regenerative responses as well as the relationship between the diameter of the microspheres and their degradation-rate, are questions that should be investigated in more detail in the future. Also, the possibility of combining fast and slow release of cells or factors by mixing alginate:collagen microspheres of different compositions and diameters may constitute an attractive method for reconstructing complex, physiological spatial and temporal concentration profiles in ischemic tissues which could prove of crucial importance for induction of a balanced angiogenic response leading to the generation of functional, stable and mature vessels rather than the dysfunctional, unstable and immature vessels found after therapeutic angiogenic induction with, for example, vascular endothelial growth factor (VEGFA) in the ischemic tissues.

## CONCLUSION

We have demonstrated the fabrication, optimization, and utilization of FGF-2, K1000 or PAECs containing hybrid alginate:collagen microspheres as delivery vehicles for pro-angiogenic treatment. The delivery of FGF-2, K1000 cells or endothelial cells at concentrations released from microspheres resulted in a dramatic increase in vascularization in zebrafish. We have also thoroughly explored the variation in scaffold properties in terms of microsphere-size, cell loading, and cell or growth factor release, by multi-factorial design allowing the synthesis of microspheres by design for future applications.

## MATERIALS AND METHODS

### Generation of alginate:collagen hydrogels

Alginate:collagen mixtures and hydrogels were produced by mixing 2% porcine collagen (Theracol) with 15 μl of 2 M NaOH (Sigma-Aldrich). This pH adjusted collagen was then mixed with 2% sodium alginate (Sigma-Aldrich) in two conjoined 10 ml luer-lock syringes, with volumes adjusted for the desired final composition. The polymer solution was cross-linked using 2% CaCl2 (Sigma-Aldrich). Degradation of the solidified hydrogels were measured using Adobe Photoshop. The degraded hydrogel particles appeared dark on the white background using phase contrast microscopy, and their density in the medium could therefore be calculated by measuring highlights (hydrogel particle-free fraction) under the select color range tool, subtract this value from the total area of the measured area of interest (particle fraction) and divide this area with the total area followed by multiplying with 100%.

### Generation of microspheres

Microspheres were synthesized via a coaxial air jet method in which micronization was controlled by regulating the applied force on the polymer solution, loaded into a single 10 ml syringe fitted with either a 27 ½ g or 30 ½ g needle (polymer flow rate), shear force (air flow rate), and the distance travelled to the coagulation bath consisting of 2% CaCl2. The coaxial air jet results in the micronization of the alginate:collagen mixture where the alginate chains are rapidly cross-linked via the ion exchange reaction between Na^+^ can Ca^2+^ upon contact with the 2% CaCl2 coagulation bath. For photography and quantification of cell encapsulation and diameter at least three separate rounds of microsphere synthesis were performed for change in synthesis parameters and at least 20 microspheres per round were analyzed.

### FGF-2 or cell encapsulation in hydrogel microspheres

After mixing collagen and alginate solutions, but prior to cross-linking, 100 μl PBS (for control samples), 100 μl FGF-2 containing K1000 conditioned medium or 100 μl of either PAEC or K1000 cells in DMEM growth media at 1.5×10^6^ or 3.0×10^6^ cells/ml were added and gently mechanically mixed. Both cell lines were a generous gift from Prof. Yihai Cao, Karolinska Institutet (Stockholm, Sweden) and recently tested negative for microsporidia. This solution was used in the microsphere synthesis setup as described above or for embedding cells in hydrogel patches. Cell laden microspheres were centrifuged at 1000 rpm for 5 min. The supernatant was removed and replaced with DI water to remove excess salt from the system. This washing procedure was repeated five times per sample. After the final centrifugation the microspheres were placed in DMEM cell growth media.

### Scanning electron microscopy

The microspheres were prepared for electron micrographs as previously described (Sherrell et al., 2016). Briefly: washed microspheres were lyophilized and sputtering with 5 nm of Au. SEM images were obtained via a LEO 1550 field-emission scanning electron microscope using an InLens detector.

### Cell sphere/tube-formation assays

One milliliter well of 1:1 ratio of alginate and collagen hydrogel was applied to the bottom of a six-well Tissue Culture Plate (SARSTEDT^®^, Nümbrecht, Germany) and cross-linked with 2% CaCl_2_. Then, 1×10^6^ PAECs/well were added onto the hydrogel followed by injection of 200 µl FGF-2 (collected from conditioned medium from culturing K1000 cells Nissen et al., 2007). PAECs analyzed for cell bodies formation were DiI labeled prior to addition as previously described. (Rouhi et al., 2010; Lee et al., 2009) Post cell addition, DMEM (HyClone™, GE Healthcare) with 10% FBS, 1% L-glutamine (HyClone™), 1% pyruvate and 1% PenStrep (DMEM growth medium) was added for incubation at 37°C with 5% CO_2_. Imaging was conducted through bright light microscopy as well as fluorescent microscopy.

### Cell viability assay

The viability of K1000 cells embedded in alginate:collagen 1:1 hydrogels were assessed using a commercial live/dead assay (Invitrogen, Thermo Fisher Scientific, cat. no L3224) according to the manufacturer's instructions. Briefly 10×10^6^ cells were mixed with hydrogel prior to hardening in CaCl2. Following hardening, cell-laden patches were washed three times and incubated in DMEM growth medium overnight and assayed for incorporation of EthD and CalceinAM by detecting OD at 530 nm (CalceinAM) and 645 nm (EthD).

### *In vivo* tolerance in mice

Microspheres consisting of 1:1 collagen and alginate of approximately 30-200 µm in diameter were centrifuged, the supernatant was removed and 100 µl of the spheres themselves were injected subcutaneously into 12, approximately 24-week-old, female C57Bl/6 mice, in a total of three technical replicates, using G27 needles on a 1 ml syringe. Five days after injection the mice were killed and the hydrogel microsphere plaques were excised and fixed in 4% PFA at 4°C overnight. Plaques were stained according to a previously described protocol with an anti-mouseF4/80 antibody (1:200, Clone CI:A3-1, AbD Serotec, Bio-Rad, Cat#: MCA497GA) to visualize macrophages, or an anti-mouseLy6G antibody (1:200, Clone RB6-8C5, Abcam, Cat#: ab25377) to visualize neutrophils, coupled to secondary goat-anti-rat-Cy5 (Millipore) or goat-anti-rabbit-Cy3 (Millipore) antibodies. The antibodies have been validated in the scientific literature as well as by the manufacturers. Animal studies were approved by the North Stockholm Research Animal Ethical Council.

### Therapeutic angiogenesis in zebrafish

PAECs and K1000 cells were injected in the perivitelline cavity of Tg(fli1a:EGFP)^y1^ (Lawson and Weinstein, 2002) transgenic endothelial reporter zebrafish embryos as previously described (Rouhi et al., 2010; Lee et al., 2009). Briefly, cells stained with 1× DiI stain (Sigma-Aldrich) for 30 min followed by multiple washing steps with PBS and re-suspended into 2×10^8^ cells/ml. Then, 48 hpf zebrafish embryos (*n*=27 divided in three technical replicates with 6-12 embryos per experiment) were manually dechorionated and anesthetized prior to injection. Microcapillaries (World Precision Instruments Inc., Florida, USA) were filled with cell suspension. Approx. 100-150 cells were injected into the perivitelline space with a microinjector (TriTech Research, Los Angeles, USA). Post injection, embryos were housed in small aqueous environment for 2-3 days followed by imaging with confocal microscopy LSM 700 (Carl Zeiss Microscopy, New York, USA). These studies were approved by the Linköping Research Animal Ethical Council.

### ELISA

We collected 100 µl of conditioned medium from hydrogel culturing at day 1,3,5 and 7 and measured with ELISA Kit KHG0021 (Invitrogen) specific for hFGF-2. Samples were read with a spectrophotometer at 455 nm (Molecular Devices, San Jose, USA) and analyzed with SoftPro Max™.

### Statistics

Samples were randomized to each experimental condition from a common group of cells, hydrogel sheets, microspheres, zebrafish embryos or mice. Treatments and quantifications of results were done in an un-blinded fashion. In all cases the *n*-values indicate the values of samples in the group with the largest variance, most often the number of colonies, sphere synthesis conditions, animals or similar, as indicated in the figure legends. All experiments were done in at least three technical replicates. The results were normally distributed and shown as means with error bars indicating the standard error, unless stated otherwise in the text. The differences in FGF-2 release from microspheres were analyzed by one-way Repeated Measures ANOVA followed by Bonferroni's post-test. A *P*-value below 0.05 was considered as statistically significant. Other statistical analysis was performed using two-tailed Student’s *t*-test, with a *P*-value below 0.05 considered to be significant. Analysis was performed using the Prism GraphPad 5 software.
